# Supplementary material for: Factors influencing wellbeing in young people during COVID-19: A survey with 6291 young people in Wales
Source: PLoS One. 2021 Dec 15;16(12):e0260640. doi: 10.1371/journal.pone.0260640 (PMC8673639; doi:10.1371/journal.pone.0260640)
Supplement: S3 File — (DOCX) [file pone.0260640.s003.docx]

**Supplementary File 3**

**Model Development: Factors considered in the model to predict emotional difficulty in the primary school years.**

Child demographical variables: gender, ethnicity, age/academic year,

Child immediate health: Got Cold/COVID, Tired, ability to concentrate

Child underlying health behaviours: Diet (positive: breakfast, fruit and veg, negative: sugary snacks, fizzy drinks, number of takeaways) , sleep duration, physical activity, number of hours sedentary, ability to ride a bike, ability to swim, travel to and from school, playtime activity, number of times brushing teeth.

Child assessment of self : Competence at school, physical literacy, general competence, behavioural difficulty.

Household/area variables: Deprivation of area, Safety, happy with area, ability to walk to a park, having a garden.

Step 1: Entering all demographic variables

| Variable | Odds ratio | 95% CI |
| --- | --- | --- |
| Female | **1.52** | **1.28 to 1.80** |
| Academic year | **0.86** | **0.78 to 0.95** |
| Asian | **0.39** | **0.22 to 0.71** |
| Black | 0.74 | 0.35 to 1.54 |
| Mixed | 1.02 | 0.73 to 1.41 |
| Not sure | 1.23 | 0.96 to 1.59 |

Step 2: Entering all immediate health variables

| Variable | Odds ratio | 95% CI |
| --- | --- | --- |
| Female | **1.51** | **1.27 to 1.80** |
| Academic year | 0.92 | 0.83 to 1.02 |
| Asian | **0.46** | **0.26 to 0.85** |
| Black | 0.75 | 0.36 to 1.61 |
| Mixed | 0.93 | 0.68 to 1.33 |
| Not sure | 1.26 | 0.97 to 1.64 |
| **Tired** | **1.44** | **1.35 to 1.52** |
| **Can concentrate** | **0.84** | **0.78 to 0.89** |
| **Got Cold/COVID** | **1.95** | **1.60 to 2.37** |

Step 3: entering all underlying health behaviours

| Variable | Odds ratio | 95% CI |
| --- | --- | --- |
| Female | **1.57** | **1.30 to 1.89** |
| **Academic year** | **0.88** | **0.79 to 0.99** |
| **Asian** | **0.46** | **0.25 to 0.85** |
| Black | 0.67 | 0.31 to 1.47 |
| Mixed | 0.98 | 0.68 to 1.39 |
| Not sure | 1.17 | 0.89 to 1.55 |
| **Tired** | **1.36** | **1.28 to 1.44** |
| **Can concentrate** | **0.88** | **0.82 to 0.95** |
| **Got Cold/COVID** | **1.91** | **1.56 to 2.35** |
| Fruit and veg | 1.02 | 0.97 to 1.07 |
| Teeth brushing | 0.84 | 0.73 to 0.98 |
| Physically active 60 minutes | 0.96 | 0.89 to 1.03 |
| Sedentary hours | 1.02 | 0.95 to 1.10 |
| **Fizzy drink consumption** | **1.14** | **1.05 to 1.22** |
| Sugary snacks | 1.01 | 0.94 to 1.09 |
| Takeaway consumption | 0.95 | 0.85 to 1.05 |
| **Ride a bike** | **0.74** | **0.58 to 0.95** |
| **Swim 25 m** | **0.77** | **0.63 to 0.95** |
| **Increasing hours of sleep** | **0.84** | **0.80 to 0.89** |
| Ran around a play time (compared to walked around) | 0.84 | 0.67 to 1.07 |
| Sat around (compared to walked around) | 1.05 | 0.71 to 1.57 |
| **Stood around (compared to walked around)** | **1.82** | **1.14 to 2.92** |
| **Played alone (compared to played with 5+)** | **4.68** | **0.28 to 7.73** |
| **Played with 1-4 others (compared to played with 5+)** | **1.53** | **1.22 to 1.92** |

Step 4: Child assessment of self-variables added

| Variable | Odds ratio | 95% CI |
| --- | --- | --- |
| **Female** | **1.91** | **1.56 to 2.34** |
| **Academic year** | **0.79** | **0.70 to 0.89** |
| **Asian** | **0.47** | **0.25 to 0.88** |
| Black | 0.52 | 0.23 to 1.21 |
| Mixed | 1.04 | 0.72 to 1.50 |
| Not sure | 1.12 | 0.84 to 1.50 |
| **Tired** | **1.30** | **1.22 to 1.39** |
| Can concentrate | 1.03 | 0.95 to 1.11 |
| **Got Cold/COVID** | **1.81** | **1.46 to 2.26** |
| Fruit and veg | 1.03 | 0.97 to 1.08 |
| **Teeth brushing** | **0.85** | **0.73 to 0.99** |
| Physically active 60 minutes | 1.01 | 0.93 to 1.09 |
| Sedentary hours | 0.98 | 0.90 to 1.06 |
| Fizzy drink consumption | 1.10 | 1.02 to 1.19 |
| Sugary snacks | 0.99 | 0.91 to 1.07 |
| Takeaway consumption | 0.91 | 0.81 to 1.01 |
| Ride a bike | 0.83 | 0.63 to 1.08 |
| Swim 25 m | 0.83 | 0.67 to 1.03 |
| **Increasing hours of sleep** | **0.88** | **0.83 to 0.93** |
| Ran around | 0.84 | 0.66 to 1.08 |
| Sat around | 0.92 | 0.60 to 1.41 |
| **Stood around** | **1.70** | **1.03 to 2.81** |
| **Played alone** | **3.72** | **2.18 to 6.35** |
| **Played with 1-4** | **1.34** | **0.23 to 0.63** |
| **Behavioural difficulty score increasing** | **1.30** | **1.24 to 1.37** |
| **School competence** | **0.78** | **0.68 to 0.89** |
| Autonomy | 0.92 | 0.82 to 1.04 |
| **General Competence** | **0.73** | **0.65 to 0.82** |
| **Physical literacy** | **0.85** | **0.75 to 0.98** |

Step 5: Local area variables

| Variable | Odds ratio | 95% CI |
| --- | --- | --- |
| **Female** | **1.86** | **1.51 to 2.28** |
| **Academic year** | **0.78** | **0.71 to 0.90** |
| **Asian** | **0.44** | **0.23 to 0.84** |
| Black | 0.52 | 0.23 to 1.22 |
| Mixed | 0.94 | 0.65 to 1.37 |
| Not sure | 1.08 | 0.81 to 1.46 |
| **Tired** | **1.28** | **1.2 to 1.37** |
| **Can concentrate** | **1.05** | **0.97 to 1.14** |
| **Got Cold/COVID** | **1.77** | **1.42 to 2.21** |
| Fruit and veg | 1.03 | 0.98 to 1.09 |
| Teeth brushing | 0.88 | 0.76 to 1.04 |
| Physically active 60 minutes | 1.02 | 0.94 to 1.10 |
| Sedentary hours | 0.98 | 0.91 to 1.07 |
| **Fizzy drink consumption** | **1.09** | **1.00 to 1.18** |
| Sugary snacks | 0.99 | 0.92 to 1.08 |
| Takeaway consumption | 0.91 | 0.82 to 1.12 |
| Ride a bike | 0.86 | 0.66 to 1.14 |
| Swim 25 m | 0.89 | 0.71 to 1.10 |
| **Increasing hours of sleep** | **0.88** | **0.84 to 0.94** |
| Ran around | 0.88 | 0.68 to 1.13 |
| Sat around | 0.91 | 0.59 to 1.40 |
| **Stood around** | **1.75** | **1.05 to 2.93** |
| **Played alone** | **3.19** | **1.85 to 5.49** |
| **Played with 1-4 others** | **1.33** | **1.04 to 1.70** |
| **Behavioural difficulty score increasing** | **1.28** | **1.2 to 1.35** |
| **School competence** | **0.81** | **0.71 to 0.93** |
| Autonomy | 0.95 | 0.84 to 1.08 |
| **General Competence** | **0.75** | **0.67 to 0.85** |
| **Physical literacy** | **0.86** | **0.76 to 0.99** |
| Safety rating of area | 0.94 | 0.86 to 1.02 |
| Safe playing area | 0.67 | 0.42 to 1.09 |
| **Happy with area** | **0.61** | **0.44 to 0.85** |
| Play all areas | 1.12 | 1.0 to 1.26 |
| Garden | 1.26 | 0.77 to 2.09 |
| Welsh Index of multiple deprivation | Most deprived 1.03  Less deprived 1.23  Low deprived 1.06  Compared to least deprived | 0.74 to 1.43  0.94 to 1.5  0.79 to 1.4 |

Step 6 : Removing non-significant variables from the model (in order of least significance eg. In order of sugary snacks, physical activity, sedentary time, autonomy, garden, swim, ride a bike, can concentrate, teeth brushing, fruit and veg, safety rating of area, WIMD.

| **Variable** | **Odds ratio** | **95% CI** |
| --- | --- | --- |
| **Female** | **1.93** | **1.59 to 2.34** |
| **Academic year** | **0.79** | **0.70 to 0.88** |
| **Asian** | **0.46** | **0.23 to 0.80** |
| Black | 0.63 | 0.29 to 1.39 |
| Mixed | 1.00 | 0.70 to 1.42 |
| Not sure | 1.11 | 0.84 to 1.48 |
| **Tired** | **1.26** | **1.19 to 1.35** |
| **Got Cold/COVID** | **1.70** | **1.37 to 2.08** |
| **Fizzy drink consumption** | **1.12** | **1.04 to 1.20** |
| **Increasing hours of sleep** | **0.90** | **0.84 to 0.94** |
| Ran around | 0.85 | 0.67 to 1.09 |
| Sat around | 0.86 | 0.57 to 1.31 |
| **Stood around** | **1.63** | **1.00 to 2.65** |
| **Played alone** | **2.99** | **1.80 to 4.96** |
| **Played with 1-4 others** | **1.29** | **1.00 to 1.61** |
| **Behavioural difficulty score increasing** | **1.27** | **1.23 to 1.35** |
| **School competence** | **0.85** | **0.75 to 0.96** |
| **General Competence** | **0.77** | **0.69 to 0.86** |
| **Physical literacy** | **0.83** | **0.73 to 0.93** |
| **Safe play area** | **0.49** | **0.39 to 0.60** |
| **Happy with area** | **0.63** | **0.47 to 0.84** |
| **Play all places** | **1.13** | **1.02 to 1.25** |
| **Takeaway** | **0.89** | **0.80 to 0.98** |

Model development - Factors considered in the model to predict anxiety in the teenage years.

Demographical variables: gender, ethnicity, age,

COVID related experience: Got Cold/COVID, had contact with someone with COVID, wore a mask yesterday, number of people meet yesterday (but no physical contact, mean= 1.9 (stdev 3.4), median= 0, max 10, min 0 ), number of people had physical contact with yesterday (mean= 0.8 (stdev 2.1), median= 0, max 10, min 0).

Household/area variables: Adults at home, having a garden.

Step 1: Entering all demographic variables

| Variable | Odds ratio | 95% CI |
| --- | --- | --- |
| **Female** | **1.20** | **1.02 to 1.41** |
| Age | 1.04 | 0.99 to 1.1 |
| Asian | 1.59 | 0.76 to 3.33 |
| Black | 0.17 | 0.02 to 1.55 |
| **Mixed** | **4.60** | **1.48 to 14.4** |
| Not reported | 3.0 | 0.53 to 16.2 |

Step 2: Entering COVID related variables

| Variable | Odds ratio | 95% CI |
| --- | --- | --- |
| **Female** | **1.21** | **1.03 to 1.42** |
| Age | 1.03 | 0.97 to 1.11 |
| Asian | 1.57 | 0.75 to 3.30 |
| Black | 0.16 | 0.02 to 1.45 |
| **Mixed** | **4.50** | **1.42 to 14.23** |
| Not reported | 2.92 | 0.53 to 16.22 |
| COVID contact | 1.29 | 0.53 to 3.12 |
| Have a cold | 0.97 | 0.540 to 1.75 |
| Wore mask yesterday | 1.36 | 0.91 to 2.04 |
| Contacts socially distanced | 0.96 | 0.89 to 1.02 |
| Direct contacts | 1.02 | 0.91 to 1.14 |

Step 3: Household level variables

| Variable | Odds ratio | 95% CI |
| --- | --- | --- |
| **Female** | **1.20** | **1.02 to 1.41** |
| Age | 1.03 | 0.97 to 1.09 |
| Asian | 1.62 | 0.77 to 3.42 |
| Black | 0.15 | 0.02 to 1.39 |
| **Mixed** | **4.37** | **1.37 to 13.93** |
| Not reported | 2.93 | 0.53 to 16.22 |
| COVID contact | 1.25 | 0.51 to 3.04 |
| Have a cold | 0.98 | 0.54 to 1.75 |
| Wore mask yesterday | 1.38 | 0.91 to 2.08 |
| Contacts socially distanced | 0.96 | 0.89 to 1.02 |
| Direct contacts | 1.02 | 0.91 to 1.14 |
| Number of adults in household | 0.92 | 0.76 to 1.10 |
| Garden | 0.94 | 0.49 to 1.84 |

Removing non-significant variable in the following order – got Cold, garden, number of physical contacts, COVID contact, number of adult in household, age, number of social distanced contacts, wore mask yesterday.

| Variable | Odds ratio | 95% CI |
| --- | --- | --- |
| **Female** | **1.23** | **1.07 to 1.4** |
| Asian | 1.44 | 0.72 to 2.89 |
| Black | 0.20 | 0.03 to 1.66 |
| **Mixed** | **5.14** | **1.68 to 15.79** |
| Not reported | 1.80 | 0.49 to 6.59 |
